# Photoinduced Spin Polarization of a Gadolinium Complex

**DOI:** 10.1021/jacs.5c20047

**Published:** 2026-03-11

**Authors:** Jonathon I. Clark, Kevin Henbest, Damyan Frantzov, Ana Štuhec, Daniel Kovacs, Ashley J. Redman, Christiane R. Timmel, Stephen Faulkner

**Affiliations:** † Chemistry Research Laboratory, Department of Chemistry, 6396 University of Oxford, Oxford OX1 3TA, United Kingdom; ‡ Centre for Advanced Electron Spin Resonance (CAESR), Inorganic Chemistry Laboratory, Oxford OX1 3QR, United Kingdom

## Abstract

This paper explores the interaction between a photoexcited organic chromophore and a gadolinium­(III) ion in a complex, which exhibits an intriguing time evolution of the spin polarization in its electron paramagnetic resonance (EPR) signature. Time-resolved EPR, transient absorption, and photoluminescence spectroscopies combine with results from density functional theory and spectral simulations to allow elucidation of the photochemical mechanism and its impact on the complex’s magnetic properties. We show that perturbation of the polarization of a gadolinium­(III) ion is possible via photoexcitation of a neighboring, organic chromophore, reflected in the inversion of the lanthanide ion’s EPR signal. We, therefore, suggest a new avenue to initialize and manipulate lanthanide-based qudits with potential applications in quantum information science.

## Graphical abstract

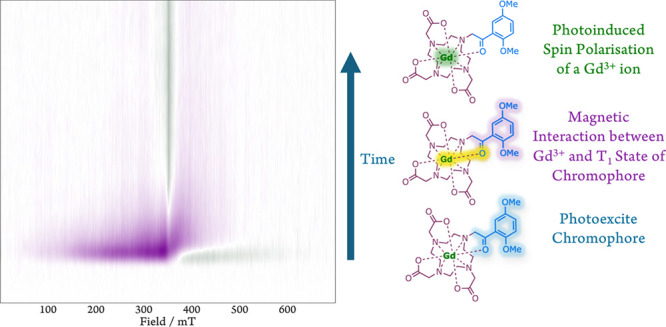

The electronic and magnetic properties of lanthanide (Ln) elements have been widely exploited in both medicine and industry, especially in the development of superconductors, laser crystals, and magnetic resonance imaging contrast agents.^1–3^ The contracted nature of the 4f orbitals and the Laporte forbidden nature of 4f–4f transitions allow Ln^3+^ ions to form non-reactive, high-spin excited states exhibiting long lifetimes.^4–6^

Higher dimension qubits, known as qudits, have become key targets in quantum information science (QIS), due to their ability to reduce the complexity of quantum algorithms and handle error correction protocols, key issues in the development of scalable quantum computing technologies.^7–11^ Molecular spin centers with *S* > 1/2 offer a versatile platform for qudit implementation, combining access to multiple quantum states with the synthetic tunability of molecular systems.^12–16^ Here, molecular lanthanide systems have gained attention, benefiting from environmental robustness due to the core-like nature of the 4f-electrons, and often relatively long coherence times, enabling coherent spin control even at relatively high temperatures.^7,17–23^

One of the key challenges of employing molecular qubits based on electron (or indeed nuclear) spins is initialization of the system into a well-defined quantum state.^24^ Boltzmann statistics for these systems typically dictate the use of both extremely low temperatures and very high magnetic fields to achieve the necessary spin polarization. An attractive strategy to form electron spin states, with up to 100% polarization, is to employ photogenerated/photoexcited molecular spin systems.^25,26^ This concept has been successfully employed across a variety of different molecules, including both organic and transition-metal complexes.^27–34^ However, because of a number of technical and conceptual challenges, this powerful approach has not yet been applied to lanthanide complexes.

Gadolinium­(III), in particular, shows exciting potential for applications in QIS, as it has the highest ground spin state, *S* = ^7^/_2_, for a single ion and no net orbital angular momentum.^21^ However, unlike for other lanthanides, direct photoexcitation is difficult due the lack of crystal field splitting of the 4f orbitals and the spin forbidden nature of the transition.^35,36^ Indirect photoexcitation, via the antenna effect, is also technically challenging due to the large energy gap (around 32000 cm^–1^) to the next excited state.^37,38^ Hence, new ways of generating excited states and manipulating the polarization of spin states of gadolinium, without using high energy excitation, could prove useful for developments in quantum information applications.

In this article, we introduce an attractive new approach to tackle this challenge by exploiting the interactions between gadolinium and a transient, excited state of a ligand chromophore.

Figure 1 introduces the core features of our approach, which allows us to manipulate the magnetic properties of a gadolinium ion, bypassing the challenge of the spin and Laporte forbidden nature of the 4f–4f transitions: instead of photoexciting a Gd^3+^ ion directly, an adjacent chromophore, **C**, is photoexcited which subsequently undergoes fast intersystem crossing (ISC) to a spin-polarized triplet state (^**3**^ **C**). We set out to investigate what effect, if any, the magnetic interaction of the ^**3**^ **C** state and the Gd^3+^ ion would have on the Gd^3+^ sublevel polarizations.

**Figure 1:**
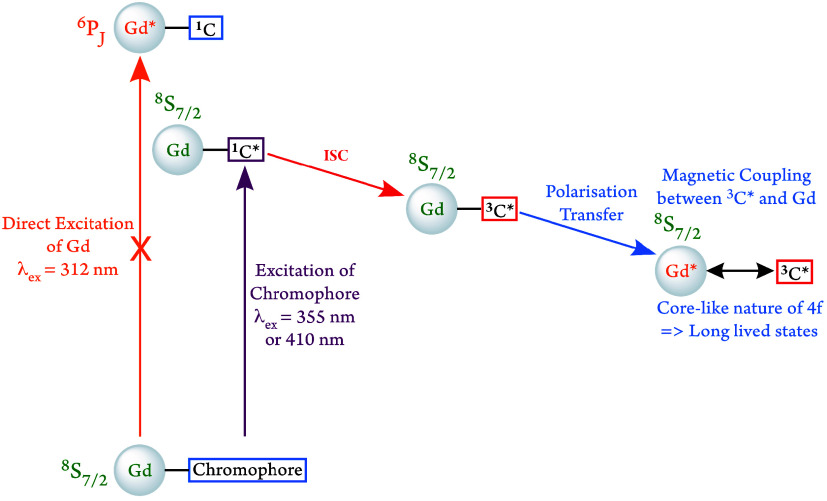
A simplified schematic detailing the possible excited state manifold and pathways for communication in a chromophore bearing the Gd^3+^ complex. All relaxation pathways and optical relaxation to ground state are ignored here.

As such, our approach circumvents the inefficient photoexcitation of the Gd^3+^ ion, but instead centers around covalently attaching a suitable chromophore, with a high triplet yield, to a kinetically robust gadolinium­(III) complex of well-defined structure. The 2,5-dimethoxyphenacyl (**DM**) chromophore (Figure 2A), was chosen for this study due to (1) its resistance to photobleaching and (2) its high triplet yield, generated by efficient ISC in keeping with El Sayed’s rule typically applicable for aryl ketones.^39,40^ The ketone group also fills the eighth coordination site of the lanthanide ion, allowing for a relatively symmetric coordination environment.

**Figure 2:**
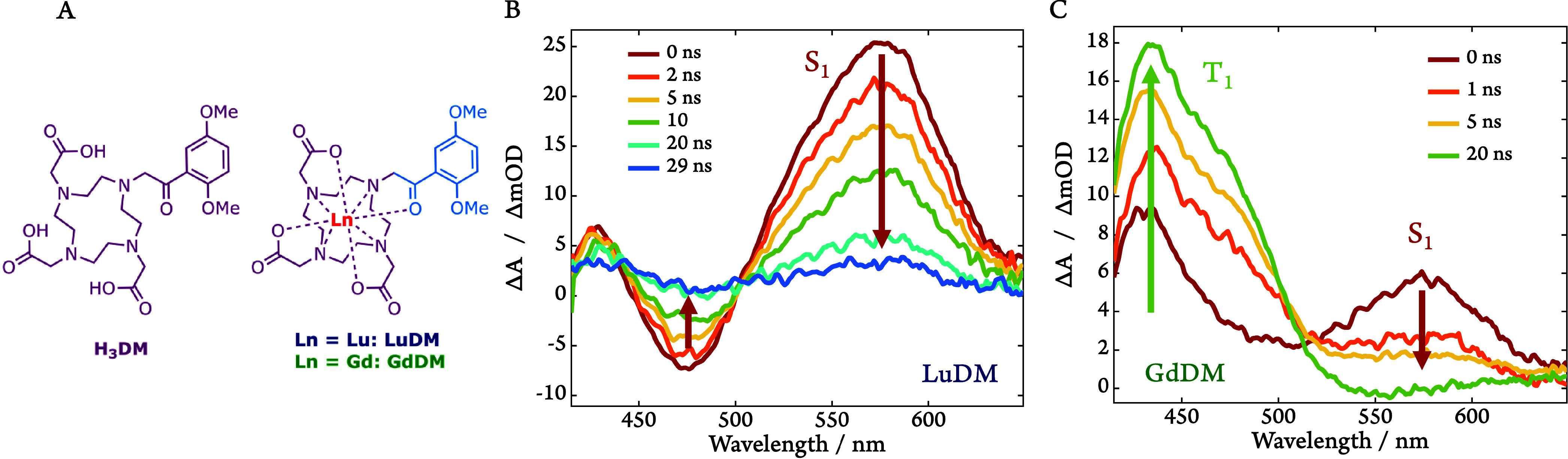
(A) Chemical structures of **H**_**3**_ **DM** and **LnDM** (Ln = **Gd**, **Lu**) introducing the color scheme valid throughout this paper. (B, C) Transient absorption spectra of **LuDM** (B) and **GdDM** (C), following photoexcitation at 410 nm (200 K). The weak emissive band (ca. 475 nm) at early times observed in panel (B) is assigned to stimulated emission (cf. Figure 3A).

**DM** was incorporated into **H**_**3**_ **DM**, which utilizes a 1,4,7,10-tetraazacyclododecane-1,4,7-triacetic acid, **DO3A**, derived lanthanide binding site. This ensured that the chromophore was in close proximity to the lanthanide ion and allowed for the possibility of strong interaction between the metal and the chromophore excited state manifold. **H**_**3**_ **DM** was synthesized following a literature procedure, and the gadolinium complex, **GdDM**, was prepared by reaction with gadolinium­(III) trifluoromethylsulfonate in a 1:1 solution of water:ethanol.^40^ The analogous lutetium complex **LuDM** was prepared in the same manner to allow direct comparison with a diamagnetic analogue. The structures of **H**_**3**_ **DM**, **LuDM**, and **GdDM** are shown in Figure 2A.

To fully characterize the photochemistry and spin polarization of the complex, we employed a combination of electron paramagnetic resonance (EPR), picosecond transient absorption (psTA), and photoluminescence (PL) spectroscopies to investigate the interaction between the excited state of the **DM** chromophore and a Gd^3+^ ion. All measurements were recorded in 7:3 glycerol:water mixtures, which form optical glasses at low temperatures.

In order to gain detailed insights into the photomechanisms of the complexes, we first conducted psTA spectroscopy on **LuDM** at 200 K to characterize the **DM** chromophore in the absence of a paramagnetic metal (see Figure 2B). Photoexcitation at 410 nm results in a broad excited state species with an absorptive maximum at 575 nm and an emissive minimum at 475 nm. As both features exhibit the same relatively short lifetime, *τ* = 13.0 ± 0.2 ns, we assigned this optical signature to a singlet state (S_1_)^41^ ascribing the negative signal around 475 nm to stimulated emission from the S_1_ state (cf. Figure 3A).

Figure 2C displays the psTA spectra for the corresponding gadolinium­(III) complex, **GdDM**. While the S_1_ band at 575 nm is still present, its intensity and lifetime, *τ* = 2.7 ± 0.2 ns for **GdDM** compared to 13 ns for **LuDM**, are much decreased. There is also another excited state species present with a maximum at 432 nm with a lifetime of *τ* = 487 ± 8 μs. The rate of formation for the species at 432 nm, τ = 2.3 ± 0.1 ns, matches closely the rate of decay for the species at 575 nm (cf. Figures S5 and S6 in the Supporting Information (SI)). This, along with a clear isosbestic point at 515 nm, suggests that the species at 432 nm may reasonably be assigned to the triplet state (T_1_). Both the decreased lifetime of the S_1_ state and increased quantum yield of the triplet state indicate that the presence of the paramagnetic Gd^3+^ cation accelerates the rate of intersystem crossing from S_1_ to T_1_.

To further investigate any differences in the photophysics of the molecules, PL spectroscopy was used to characterize any emissive radiative processes (Figure 3A). Under continuous excitation at 405 nm, all three complexes exhibit a single emissive band centered at 475 nm (**H**_**3**_ **DM)** and 495 nm (**GdDM and LuDM**), respectively. The red shifting of the emission band for the metal complexes is ascribed to the effect of coordinating a highly Lewis-acidic Ln^3+^ ion to the chromophore (also see their UV/vis absorption spectra in Figure S4 in the SI).

**Figure 3:**
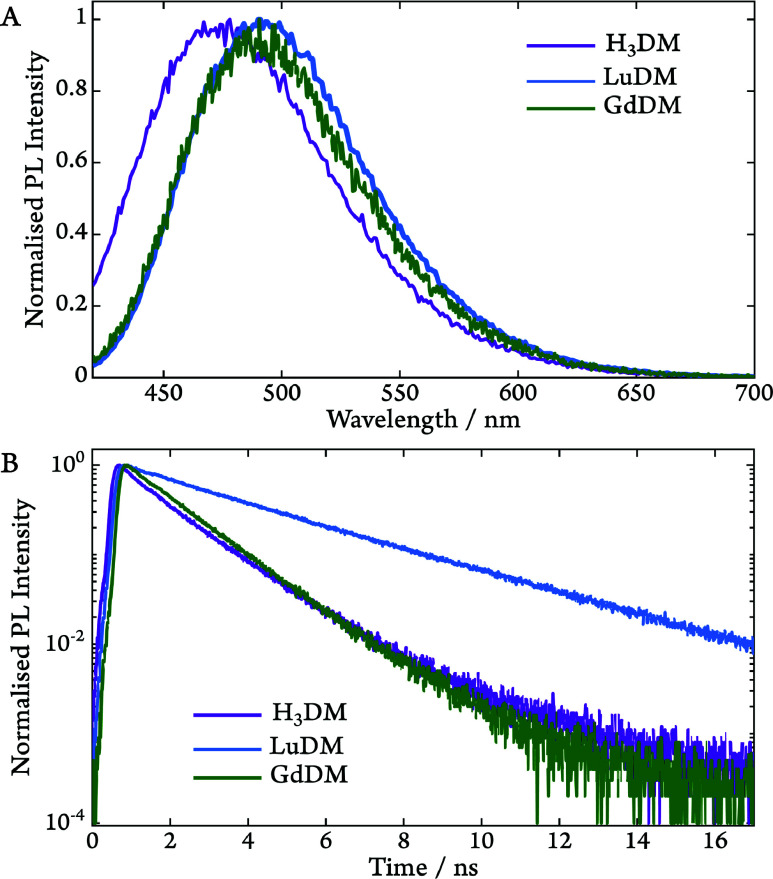
(A) Normalized emission spectra of **H**_**3**_ **DM** (purple), **LuDM** (blue), and **GdDM** (green). (B) TCSPC kinetics for **H**_**3**_ **DM** (purple), recorded at 475 nm, and **LuDM** (blue), recorded at 492 nm, and **GdDM** (green), recorded at 495 nm, see Figures S8 and S9 in the SI for data fitting including the Instrument Response Function. All data were obtained at 300 K and λ_ex_ = 405 nm.

To extract the lifetimes of the radiative processes, time-correlated single-photon counting (TCSPC) was employed, as shown in Figure 3B. The lifetimes of all three complexes in Table 1 suggest the observed emission bands are likely due to fluorescence from the S_1_ state. A couple of observations can be made. First, in keeping with the psTA data above, inserting the Gd^3+^ ion into the structure results in a shortening of excited singlet state lifetime. Second, the lifetime of **LuDM** exceeds that of **H**_**3**_ **DM**, likely due to a subtle change in geometries of the ground and excited states of the chromophore upon coordinating the Ln^3+^ ion. Crucially, this proves that a heavy atom effect can be dismissed as the origin of the decrease in the lifetime of the S_1_ state for **GdDM**. Hence, to further investigate the intersystem crossing and the nature of the T_1_ state, time-resolved EPR (trEPR) spectroscopy was performed.

**Table 1:** Emission lifetime Data (300 K, λ_ex_ = 405 nm), extracted from TCSPC measurements of **H**_**3**_ **DM**, **LuDM** and **GdDM** (Figures S8 and S9 in the SI)­^a^

Compound	Lifetime (ns)
**H**_**3**_ **DM**	1.63 ± 0.09
**LuDM**	3.47 ± 0.09
**GdDM**	1.39 ± 0.11

a The emission intensity was recorded at 475 nm (**H**_**3**_ **DM**), 492 nm (**LuDM**), and 495 nm (**GdDM**), respectively.

Figures 4A and 4B report the trEPR spectra of **H**_**3**_ **DM** and **LuDM**, obtained following a 410 nm, 5 ns laser pulse. The close resemblance of the data on inspection, including their similar polarisation pattern and spectral width, is due to the shared origin of the complexes’ triplet states on the **DM** chromophore, resulting in similar zero-field splitting (ZFS) parameters, *D* and *E*, and relative [*p*_*x*_:*p*_*y*_:*p*_*z*_] sublevel populations. This conclusion is supported by density functional theory (DFT), which is used to provide initial estimations of the spin Hamiltonian parameters, from which the experimental spectra could be fitted via the esfit function of EasySpin.^42–45^ The small differences in the parameters, provided in Figure 4, are due to the slight difference in geometries of the chromophore in the two complexes. Crucially, however, the EPR signature of both complexes maintains its spin polarization pattern throughout the lifetime of the EPR signal (see Figures S10, S11, S13, and S14 in the SI), which is identical for **LuDM** and **H**_**3**_ **DM** (Figure 4C). The triplet state’s trEPR signal is much shorter lived than observed for **GdDM** (432 nm) in psTA. This is not surprising as any relaxation of the triplet sublevels to Boltzmann populations results in a loss of trEPR but not psTA signal. Finally, the data demonstrate that delocalization of spin density onto the Lu^3+^ ion is negligible, as only a minimal heavy atom effect is observed.

**Figure 4:**
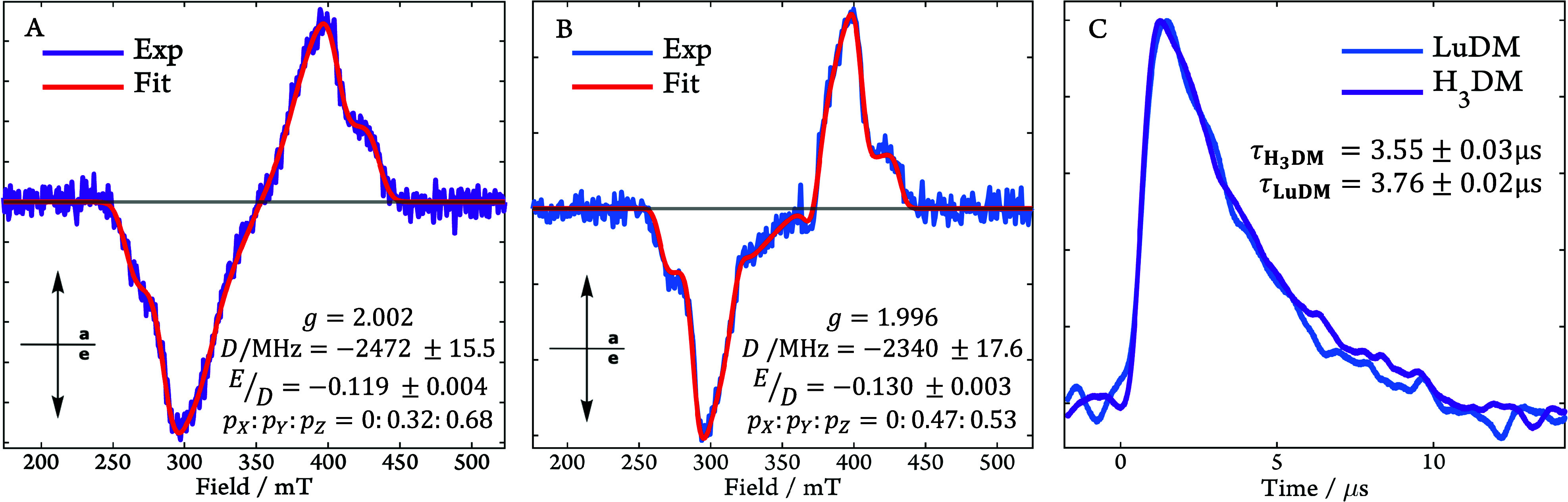
trEPR spectra recorded and averaged 1.1–1.8 μs (80 K, λ_ex_ = 410 nm) after the laser flash for (A) **H**_**3**_ **DM** and (B) **LuDM**. The insets provide the corresponding EasySpin simulation parameters. In keeping with DFT predictions, a negative sign of the ZFS parameter, *D*, was used in the spectral simulations for both species. (C) Time evolution of the trEPR signals (200 K, λ_ex_ = 410 nm) for **H**_**3**_ **DM** and **LuDM** averaged over 397–402 mT and normalized to their respective maxima in signal intensity. The lifetimes in the legend were obtained by a monoexponential fit (see SI Figure S10, S11).

Having explored the ground-state diamagnetic complexes, we turned our attention to the trEPR spectrum of **GdDM**. Figure 5A reports the time- and field-resolved EPR spectra (left) highlighting time slices obtained at early times (1–2 μs, bottom, right) and late times (7–11 μs, top, right) after the laser flash. Upon inspection, it is obvious that the EPR spectra exhibit an interesting time evolution. Roughly, there appear to be two regimes: the decay of an initial, largely absorptive, broad peak concomitant with the rise of a sharp emissive signal centered around *g* = 1.992 showing no visible decay over our measurement (16 μs).

**Figure 5:**
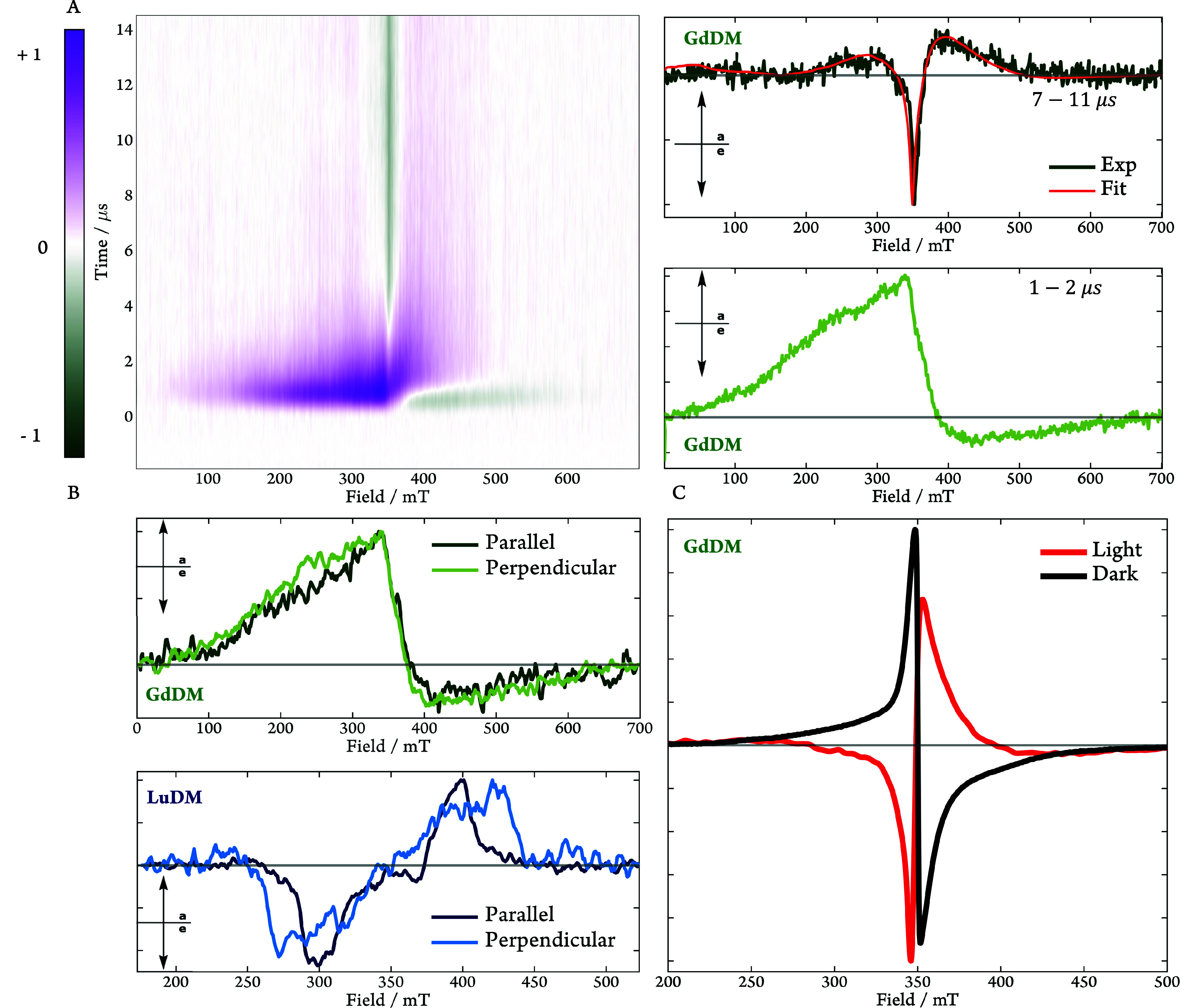
(A) trEPR spectra of **GdDM** (80 K, λ_ex_ = 355 nm). (Left) Time- and field-resolved spectra; (right) time-averaged time slices obtained 1–2 μs (bottom) and 7–11 μs (top, green) after the laser flash. The averaged spectrum recorded at late times includes a fitted spectrum (red), simulated by varying the polarization of the Gd^3+^ sublevels in the eigenbasis, while using the same spin Hamiltonian parameters extracted from fitting the cwEPR spectrum in the dark (SI Figure S21). See the main text and SI for details of the fitting procedure. B: Magnetophotoselection (MPS) data for **GdDM** (top, green, λ_ex_ = 355 nm) and **LuDM** (bottom, blue, λ_ex_ = 410 nm). Parallel/Perpendicular indicates the orientation of the polarization of incident laser radiation relative to the B_0_ field. The spectra were recorded at 80 K and were averaged between 1–2 μs after the laser flash. Note: spectra are normalized to their respective signal maxima to highlight the effects of MPS on spectral shape. (C) Experimental ground state cwEPR spectrum of **GdDM** (black) and simulation (red) of a cwEPR spectrum employing the simulation magnetic parameters obtained for the photoinduced trEPR spectrum in part A (top, right). Data in Figure S16 in the SI illustrates that the choice of wavelength (355 nm vs 410 nm) has no effect on the appearance of trEPR spectra for GdDM, apart from an improvement in S:N for λ_ex_ = 355 nm.

Following the results from the diamagnetic analogues as well as the optical spectroscopy above, the chromophore T_1_ state seems, at first, an obvious suggestion for the short-lived component but the **GdDM** trEPR spectrum bears no resemblance at all to either that of **H**_**3**_ **DM** or **LuDM**. Instead, its spectral width, exceeding some 600 mT, is reminiscent of a Gd^3+^ signature but its unusual polarization suggests that its origin lies in the direct communication between the chromophore triplet and Gd^3+^ ^8^S_7/2_ states.^46–48^

The loss of pure triplet character is further confirmed by the absence of a clear magnetophotoselection effect on the spectral shape of the initial **GdDM** trEPR spectrum (Figure 5B), typically expected for a triplet state formed via vibronic coupling, as shown by its **LuDM** counterpart. Given the complexity of the spectrum, it is, hence, not surprising that simulations have not, as of yet, returned a unique set of ZFS parameters/populations/coupling regimes. We can, however, state safely that the initially observed signal follows formation of the spin polarized chromophore T_1_ state and exhibits features arising from the complex interplay of the T_1_ and ^8^S_7/2_ states. The theoretical investigation of this complex system forms part of a much larger study in our group to be reported elsewhere.

Returning to an inspection of Figure 5A, the most striking feature is the evolution of the EPR signature from a broad featureless signal to a sharp, long-lived emissive spectrum, whose appearance is reminiscent of an “inverted” Gd^3+^ cwEPR spectrum (Figure 5A, top right). The broad trEPR signals resulting from the triplet states of **H**_**3**_ **DM** and **LuDM** both decay significantly over 16 μs, whereas this sharp, emissive signal exhibits no observable decay in signal intensity over this time window. (See Figures S13–S15 in the SI for full details.) As the first electronic excited state of Gd^3+^ lies some 4000 cm^–1^ above the employed photoexcitation energies, it can be concluded that the observed signal is due to a spin polarized Gd^3+^ ion, following polarization transfer from the T_1_ state of the chromophore.^37,38^

To shed light on the nature of the polarization, we recorded the ground-state cwEPR spectrum (Figure 5C, black), dominated by the central feature (in high fields assigned to the $-\frac{1}{2}\to +\frac{1}{2}$ transition).^46^ We undertook the simulations of both the long-lived trEPR and the ground-state cwEPR spectra (Figure S21 in the SI) adapting the approach of Clayton et al.^48^: the large anisotropic broadening, due to the strain of the ZFS parameter, *D*, was addressed through explicit sampling of a bi-modal Gaussian distribution for *D*, centered at ±*D*_0_ with full width at half-maximum, *D*_fwhm_. This method accounts for deviations from axial electronic structures by explicitly sampling the other ZFS parameter, *E*, between 0 < *E* < *D*/3 for each value of *D*. The *g*-value was set to 1.992 and values for *D*_0_ = 1008 MHz and *D*_fwhm_ = 851 MHz could be obtained from the cwEPR spectrum.

The long-lived trEPR signal, which maintains its spin polarization beyond 16 μs, was then simulated (red, Figure 5A, top right), using the same values for *D*_0_ and *D*_fwhm_ as the ground state cwEPR spectrum, by only adjusting the polarization of the Gd^3+^ sublevels in the eigenbasis and the asymmetry parameter, *ASP*, which is the intensity ratio between the distributions centered at +*D*_0_:–*D*_0_. Additionally, its corresponding field-modulated derivative spectrum was subsequently generated to compare photogenerated (red) and ground state (black) (see Figure 5C). As shown in detail in the SI, this approach demonstrates that the polarization of the Gd^3+^ sublevels is altered upon photoexcitation such that the central transition is inverted. While quantitative analysis of the origin of the polarization and its evolution in this complex goes far beyond the scope of this letter, this study provides, to our knowledge, the first evidence of photogenerated spin polarization of a lanthanide ion.

In conclusion, Gd­(III) and Lu­(III) complexes containing the 2,5-dimethoxyacetphenone chromophore were synthesized and photophysically characterized. Photoexcited states of the lanthanide complexes were explored by trEPR demonstrating a photogenerated inversion of the Gd^3+^ ion EPR signal, the first evidence of photoinduced spin polarization of a lanthanide ion. With further extensive studies elucidating the mechanisms of spin–spin interaction, energy and spin polarization transfer underway in our group, this work provides an exciting new avenue of initializing lanthanide complexes-based, molecular spin qudits.

## Supplementary Material



## Notes

Experimental methods; synthetic and sample preparation protocols; characterization by NMR and mass spectrometry; additional EPR and optical spectroscopy data; results of DFT calculations; EPR data analysis methods; fitting parameters and additional details on spectral simulations (PDF)
