# Supplementary material for: Photoinduced Spin Polarization of a Gadolinium Complex
Source: J Am Chem Soc. 2026 Mar 11;148(11):11438–44. doi: 10.1021/jacs.5c20047 (PMC13022879; doi:10.1021/jacs.5c20047)
Supplement: Supplementary file 1 [file ja5c20047_si_001.pdf]

Photoinduced Spin Polarisation of a Gadolinium Complex

SUPPLEMENTARY INFORMATION

Jonathon I. Clark[1,2], Kevin Henbest[1], Damyan Frantzov[1], Ana Štuhec[1], Daniel Kovacs[1], Ashley J. Redman[1,2], Christiane R. Timmel[1,2 *] and Stephen Faulkner[1*]

[1]Chemistry Research Laboratory, Department of Chemistry, University of Oxford, OX1 3TA

[2]Centre for Advanced Electron Spin Resonance (CAESR), Inorganic Chemistry Laboratory, OX1 3QR

1. Preparation of Complexes ***3***

a. Synthesis H_3-DO3A-2,5-dimethoxyacetophenone, H_3DM **3**

b. Synthesis of Ln-DO3A-2,5-dimethoxyacetophenone, LnDM **3**

2. Chemical Characterisation ***3***

a. Nuclear Magnetic Resonance (NMR) **3**

NMR of LuDM 4

b. Mass Spectrometry (MS) **5**

LuDM 5

GdDM 5

3. Sample Preparation for Spectroscopic Investigations Below ***6***

4. Optical Spectroscopy ***6***

a. UV/Vis **6**

b. Picosecond Transient Absorption (psTA) Spectroscopy **6**

Experimental Considerations 6

Lifetime Measurements 6

c. Photoluminescence (PL) Spectroscopy: **7**

General Experimental Considerations 7

Emission Spectra 7

Excitation Spectra 7

Time Correlated Single Photon Counting (TCSPC) 8

5. Electron Paramagnetic Resonance (EPR) Spectroscopy ***9***

a. General Procedure **9**

b. Continuous Wave Measurements **9**

c. Time resolved Measurements **9**

200 K trEPR Data 9

80 K trEPR Data 10

Additional trEPR Experiments 12

6. Density Functional Theory (DFT) ***12***

a. Spin Density Plots **13**

7. Spectral Simulations ***14***

a. H_3DM **14**

b. LuDM **15**

b. GdDM **15**

Simulation Procedure 15

Spin Hamiltonian Parameters 16

Evaluation of the Asymmetry Parameter 17

Comparison to Other Similar Gd^{3+} ions 17

Comparison of Light vs Dark Spectra 18

References ***20***

1. Preparation of Complexes

a. Synthesis H_3-DO3A-2,5-dimethoxyacetophenone, H_3DM

H_3-DO3A-2,5-dimethoxyacetophenone, **H_3DM**, was synthesised according to a known literature procedure.[1]

b. Synthesis of Ln-DO3A-2,5-dimethoxyacetophenone, LnDM

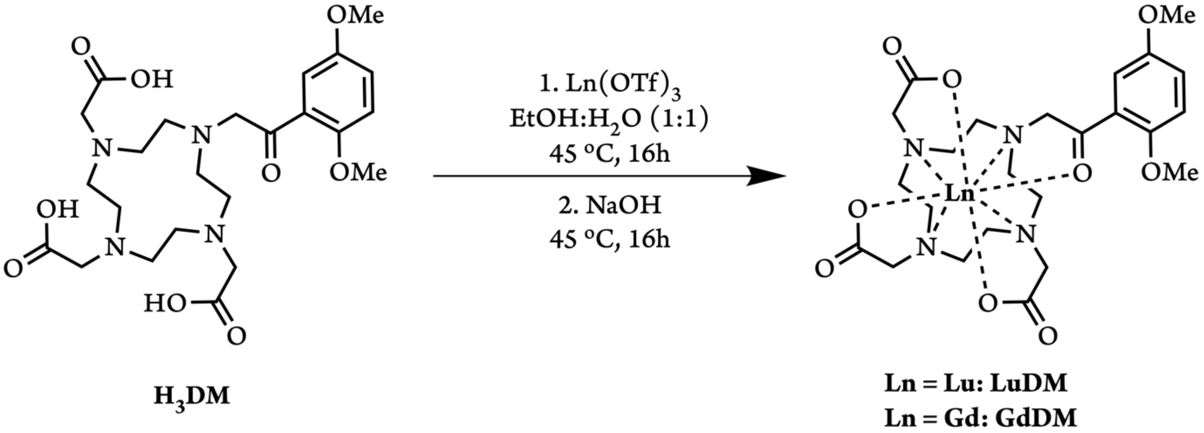

Scheme 1: Synthesis of **LnDM** from **H_3DM**.

To a solution of **H_3DM** (100 mg, 0.200 mmol) in 1:1 EtOH:H_2O (1.9 mL), the appropriate lanthanide triflate salt (Ln = Gd: 125 mg, Ln = Lu: 127 mg, 0.191 mmol) was added and the reaction mixture was stirred at 40 °C overnight. The pH was adjusted to pH = 6–7 by dropwise addition of an aqueous 1 M NaOH solution (0.614 mL, 0.614 mmol) and left stirring overnight. The solution was then filtered, and solvent was removed *in-vacuo*. The solution was purified via dialysis in a 500 M dialysis chamber. The solvent was then removed *in-vacuo* before the product was dried under high-vacuum for 48 h to leave a yellow powder.

2. Chemical Characterisation

a. Nuclear Magnetic Resonance (NMR)

AVH400 (400MHz) is a Bruker Avance III HD nanobay NMR equipped with a 9.4 T magnet. ^{1}H 400.1MHz, ^{13}C 100.6MHz. ^{1}H and ^{13}C NMR spectra were internally referenced to residual protio-solvent (^{1}H) or solvent (^{13}C) resonances and are reported relative to $SiMe_4$ (δ = 0 ppm). Chemical shifts (δ) are quoted in ppm and coupling constants in Hz.

NMR of LuDM

^{1}H NMR (400 MHz; d^{2} -D_2O, 298 K): δ 7.49 (d, 1H, ArH, $^{4}J_{H\text{-}H}$ = 3.25 Hz), 7.42 (dd, 1H, ArH, $^{3}J_{H\text{-}H}$ = 9.32 Hz, $^{4}J_{H\text{-}H}$ = 3.25 Hz), 7.25 (d, 1H, ArH, $^{3}J_{H\text{-}H}$ = 9.32 Hz), 3.97 (s, 3H, O-CH3), 3.84 (s, 3H, O-CH3), 3.67 (b, m, 2H, Cyclen N-CH_2), 3.46 (b, m, 2H, Cyclen N-CH_2), 2.87 (b, m, 5H, Cyclen N-CH_2), 2.56 (b, m, 7H, Cyclen N-CH_2).

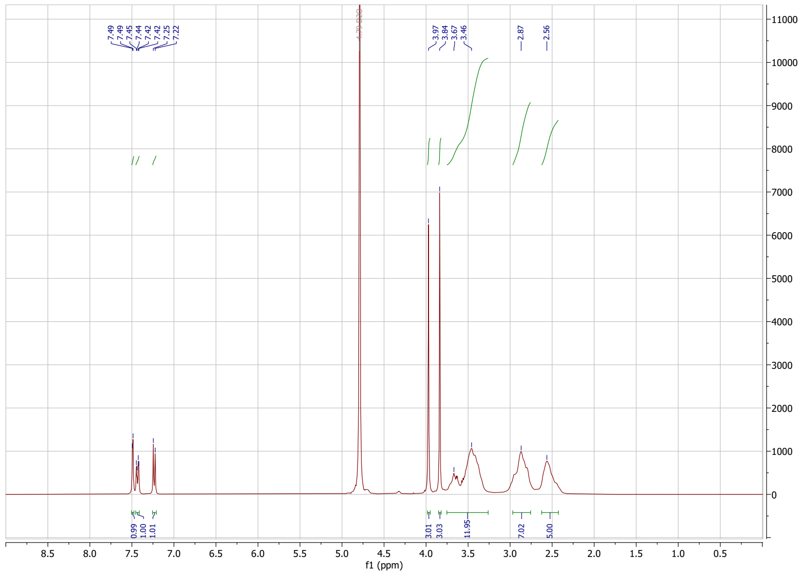

Figure S1: ^{1}H NMR spectrum of **LuDM**, d^{2}-D_2O, 298K

^{13}C NMR (400 MHz; d^{2} -D_2O, 298 K): δ 208 (C=O ketone), 181 (C(OO)-), 157 (C(OO)-), 153 (C(OO)-), 126 (Ar), 122 (Ar) , 121 (Ar) , 118 (Ar) , 115 (Ar) , 114 (Ar), 66 (Cyclen - CH2), 56 (O-CH3), 56 (O-CH3), 55-54 (Cyclen - CH2).

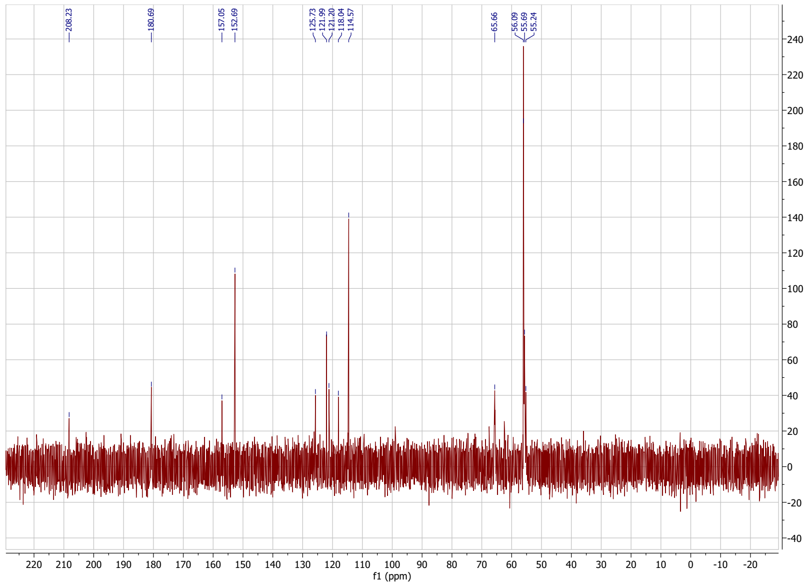

Figure S2: ^{13}C NMR spectrum of **LuDM**, d^{2}-D_2O, 298K.

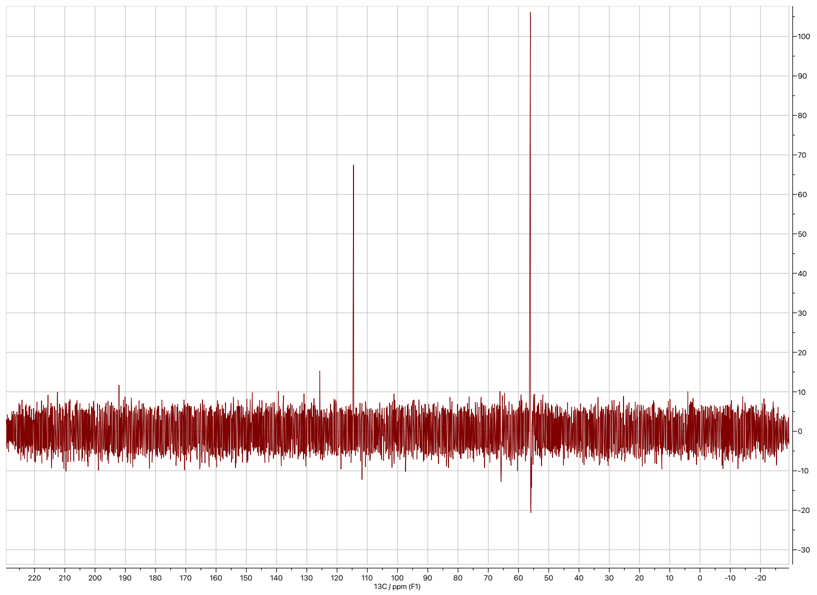

Figure S3: ^{13}C DEPT spectra of **LuDM**, d^2-D_2O, 298K

b. Mass Spectrometry (MS)

Low Resolution Mass Spectrometry (LRMS) spectra were measured using a Waters LCT Premier XE bench-top orthogonal acceleration time-of-flight LC-MS system, currently used without front end LC in direct infusion (loop injection) mode. Connected to a Waters 1525u Binary HPLC Pump and a Waters/CTC Analytics 2777C Sample Manager. High Resolution Mass Spectrometry (HRMS) were measured using a Waters RDa bench-top TOF used with an Acquity LC system in direct infusion (loop injection) mode for target confirmation.

LuDM

LRMS

Experimental: [M+Na$^+$] = 718.933 (100%), 719.940 (14.3%), 719.892 (7.92%), 719.535 (5.12%) 719.449 (4.0%). [M+H$^+$] = 696.950 (60.78%), 697.929 (7.99%), 696.885 (7.10%), 698.63 (6.73%). Theoretical: [M+Na$^+$] = 719.16 (100.0%), 720.16 (29.3%), 721.16 (6.4%), 720.15 (1.5%). [M+H$^+$] = 696.17 (100.0%), 697.17 (29.3%), 698.17 (6.3%), 697.16 (1.5%)

HRMS

Found: [M+H$^+$]= 697.1727 (Theoretical = 697.1728); Found: [M+Na$^+$]= 719.1557 (Theoretical = 719.1548).

GdDM

LRMS

Experimental: [M+H$^+$] = 680 (100.0%), 678 (82.4%), 682 (66.7%), 679 (75.9%), 677 (47.8%), 681 (30.3%). Theoretical: [M+H$^+$] = 680.15 (100.0%), 678.15 (80.5%), 682.15 (73.6%), 679.15 (72.0%), 677.15 (50.5%), 681.15 (24.2%).

HRMS

Found: [M+H$^+$]= 680.1565 (Theoretical = 680.1567); Found: [M+Na$^+$]= 702.1365 (Theoretical = 702.1381)

3. Sample Preparation for Spectroscopic Investigations Below

All samples were prepared in a 7:3 glycerol:water solutions. MiliQ water, with a resistivity of 18.2 mΩ, was used. Glycerol was purchased from Sigma Aldrich.

4. Optical Spectroscopy

a. UV/Vis

UV/Vis absorption data was recorded using a Shimadzu UV-1601 UV-vis spectrophotometer with sampled housed inside a quartz cuvette. Spectra are reported relative to a 7:3 glycerol:water baseline.

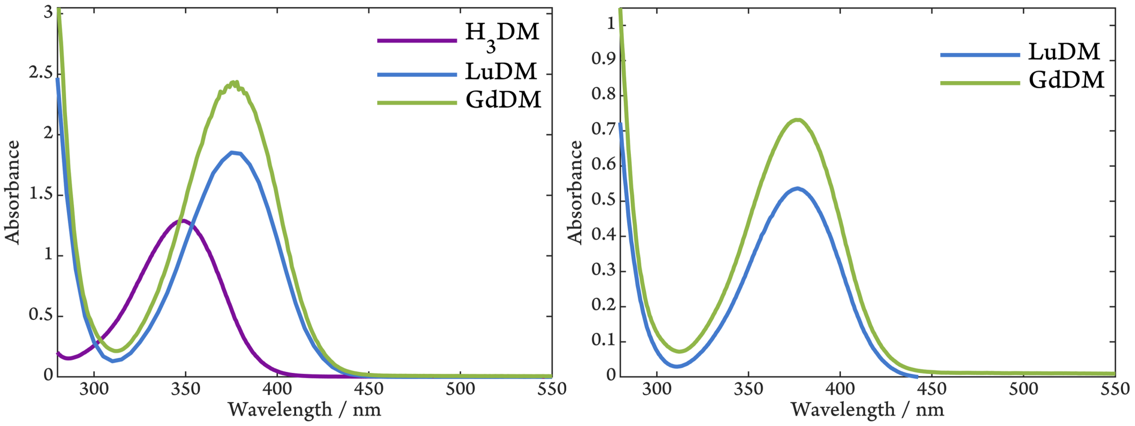

Figure S4: UV/Vis absorption spectra of **H_3DM**, **LuDM** and **GdDM** at 2mM (left), and **LuDM**, **GdDM** at 0.4 mM, (right), all at 298K.

b. Picosecond Transient Absorption (psTA) Spectroscopy

Experimental Considerations

The concentration of the sample was prepared so that absorbance at λ_{ex} = 410 nm was 0.8 relative to a pure 7:3 glycerol:water mixture. psTA spectroscopy measurements were performed on an Ultrafast Systems EOS spectrometer. Pump excitation was provided by a mode locked picosecond Nd:YAG laser and OPG (Ekspla PL2210 and PG403), with a pulse width of 15–20 ps operating at a repetition rate of 250 Hz. The OPG was pumped with 200 μJ pulses at 355 nm, resulting in an energy at the excitation wavelength (410 nm) of approximately 10 μJ per pulse. The probe light source of the spectrometer is a supercontinuum white light laser with a range of 350–900 nm at a 500 Hz repetition rate. The spectrometer incorporates a cryostat (Oxford Instruments OpistatCF) to accommodate the sample at the point of pump-probe overlap. All measurements were performed at 200 K.

Lifetime Measurements

Decay data were fitted using a custom Python script utilising a least-squares cost function. All data was fitted with a monoexponential fit. All figures show the experimental data (black) with a fit (red) overlayed.

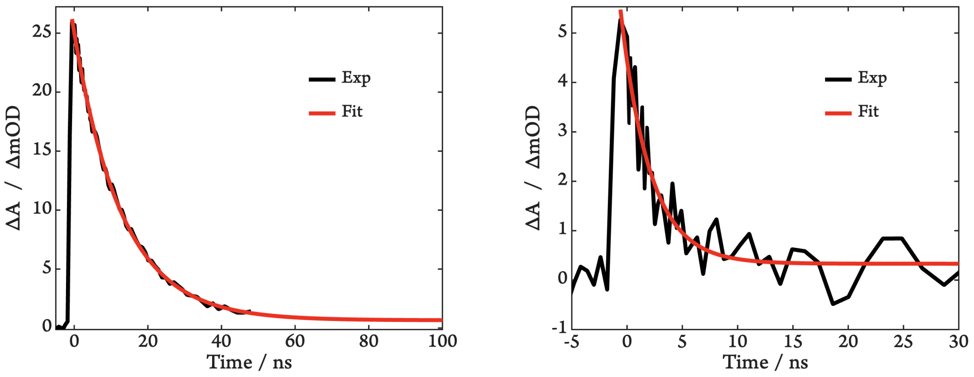

Figure S5: Decay of psTA signals of **LuDM** (left) and **GdDM** (right) at 200 K at 575 nm. Rate constants provided in main text.

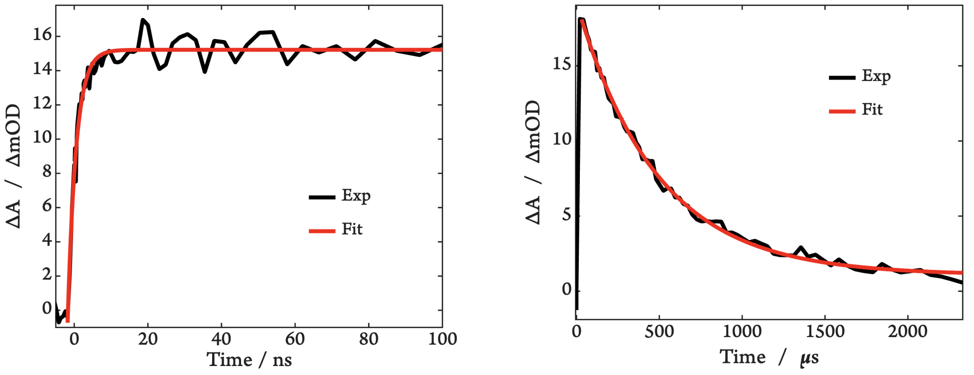

Figure S6: Formation of psTA signal of **GdDM** at 447 nm (left) and decay of psTA signal of **GdDM** at 441 nm (right), both at 200 K. Rate constant provided in main text.

c. Photoluminescence (PL) Spectroscopy:

General Experimental Considerations

All measurements were recorded on an Edinburgh Instruments FS5 Spectrofluorometer in a quartz cuvette with a 1 cm pathlength. A polariser aligned at 54.7° was placed after the sample in order to remove rotational correlation effects. The concentration of the samples was chosen such that the absorption at 405 nm was 0.05 to prevent inner filter effects. All results used an Edinburgh Instruments pulsed laser diode (EPL) at 405 nm unless otherwise stated.

Emission Spectra

Emission spectra, see main text, were recorded using a repetition rate of 20 MHz and the spectra were swept from 420–800 nm. Each spectrum was recorded three times and the results summed.

Excitation Spectra

Excitation spectra were recorded at the maximum signal intensity of the emission spectra for the respective species (**H_3DM**: 474 nm, **LuDM**: 492 nm, **GdDM**: 490 nm). Excitation spectra utilised a xenon lamp, and the spectra were swept from 300–450 nm. The **H_3DM** excitation spectrum is red shifted relative to the UV/Vis absorption spectrum by about 20 nm. This excitation spectrum shows similar photoluminescence intensity at 355 nm and 410 nm for **GdDM** despite the difference in absorbance at these two wavelengths in the UV/Vis spectrum, hence, justifying the choice of the more convenient 410 nm as the wavelength for optical spectroscopy for all complexes.

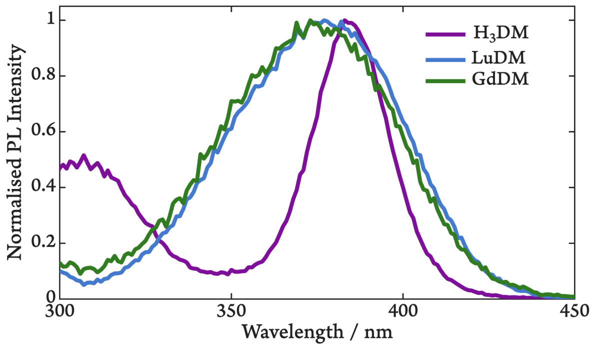

Figure S7: Excitation spectra at of **H_3DM**, **LuDM**, **GdDM** at 300 K.

Time Correlated Single Photon Counting (TCSPC)

TCSPC spectra were recorded using a repetition rate of 10 MHz and were recorded at the maximum in the emission spectra for all three complexes. All spectra were recorded until the maximum signal intensity reached 10^4 counts. The instrument response factor, IRF, is plotted (blue) in all cases and was recorded until intensity reached 10^4 counts. Tail fitting (red) started after the IRF was negligible. All simulations utilised a monoexponential fit with a small offset using a custom MATLAB script.

TCSPC of H_3DM, LuDM & GdDM

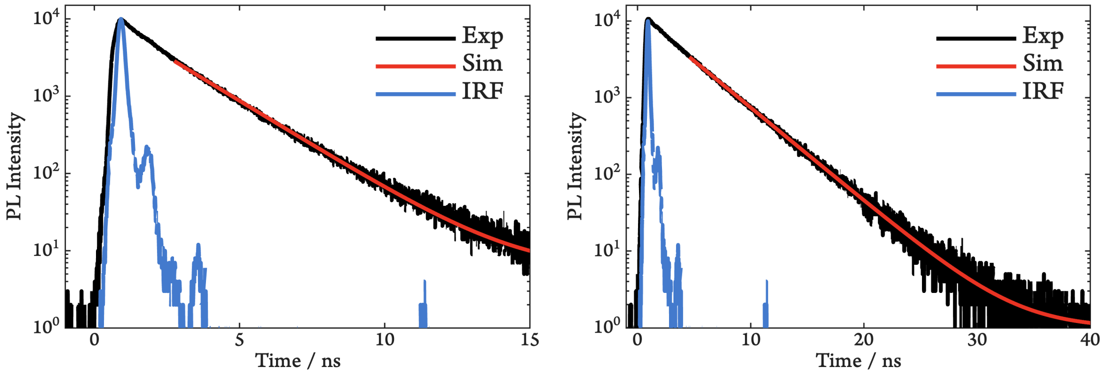

Figure S8: TCSPC decay at 470 nm of **H_3DM** (left) and at 492 nm for **LuDM** (right), both at 300 K. Rate constants provided in Table 1 in the main text.

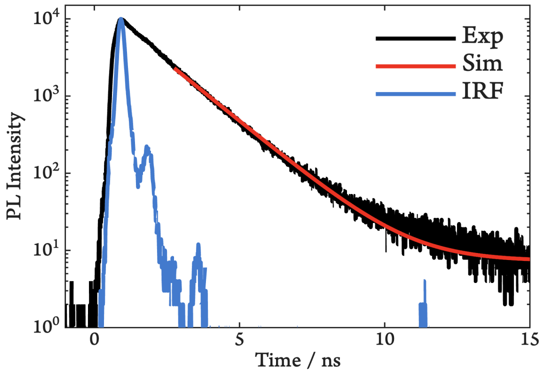

Figure S9: TCSPC decay at 492 nm of **GdDM** at 300 K. Rate constant provided in Table 1 in the main text.

5. Electron Paramagnetic Resonance (EPR) Spectroscopy

a. General Procedure

Measurements were performed at X-Band using either a Bruker E680 or E580 spectrometer at both 200 K and 80 K with critically coupled ER-4118X-MD5-W1 or EN 4118X-MD4-W1 resonators. The temperature was controlled using a nitrogen gas-flow cryostat and an ITC temperature controller from Oxford Instruments. The concentration used was 2 mM for all samples.

b. Continuous Wave Measurements

The cwEPR measurement was performed on an E580 spectrometer at X-Band. The field modulation was set to 2.000 G with a frequency of 100 kHz. The field was swept from 0–700 mT with 2048 points. The dependence of the signal intensity as a function of the microwave power was checked to ensure anisotropic broadening, as a result of saturation effects, were not prevalent.

c. Time resolved Measurements

The trEPR measurements were performed with direct detection using the CW detector with AC-AFC and a Stanford Research 560 voltage preamplifier with a 3–300 kHz bandpass. A background correction was performed in two steps: first subtracting the average signal before the laser pulse and secondly by subtracting a linear plot through averages of the signal at very high and very low field. All data were corrected to 9.75 GHz. Lifetimes were calculated by fitting a monoexponential curve (with a small offset) to the decay using the same custom MATLAB script as before. All spectra were measured with a time base of 2 ns with 8192 points. For all 2D – colour plots, purple represents absorbative signal and green emissive signals. For figures showing signal intensity vs field at different time slices, the data was normalised to the maximum signal intensity across all times and fields.

Laser excitation was performed at 410 nm for **H_3DM, LuDM** and at 355 nm for **GdDM** unless otherwise stated with an energy of 1–2 mJ per shot. The power was controlled with a λ/2 waveplate and a Glan-Taylor polariser. An achromatic depolarizer was placed after the final turning mirror, and the light was then directed into the optical window of the cryostat. The following lasers were used:

i) Opotek Opolette HE355 OPO laser pumped by the third harmonic of a Nd:YAG laser with a shot repetition rate of 20 Hz. (Opolette)

ii) EKSPLA NT230-50-SH/SF-SCU-FC-2H OPO laser pumped by the third harmonic of a Nd:YAG laser with a shot repetition rate of 50 Hz. (EKSPLA)

200 K trEPR Data

Below the trEPR of all three species at 200K together with their kinetic profile is provided.

trEPR of H_3DM

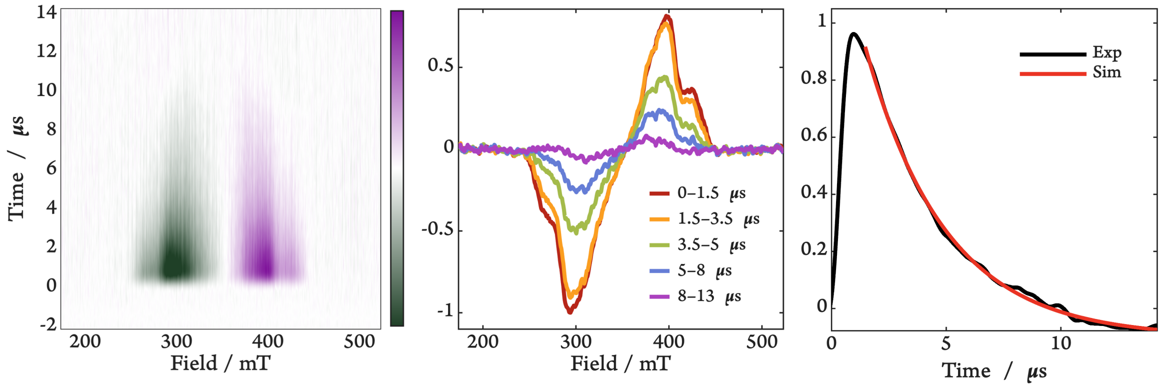

Figure S10: **H_3DM**, trEPR, 200 K, Opolette. Left: Time- and field-resolved data. Middle: Selected time slices of trEPR spectra. Right: trEPR intensity at 398 mT as shown in Figure 4C of main text (black) simulated with a monoexponential decay with a small offset (red) introduced by noise over the small field range included in the fit. Rate constants provided in Figure 4C in main text.

trEPR of LuDM

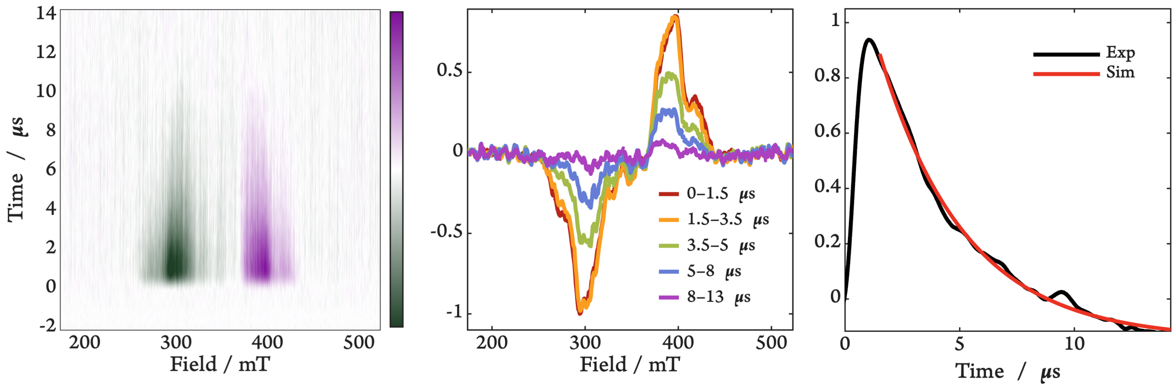

Figure S11: **LuDM**, trEPR, 200 K, Opolette. Left: Time- and field-resolved data. Middle: Selected time slices of trEPR spectra. Right: trEPR intensity at 398 mT as shown in Figure 4C of main text (black) simulated with a monoexponential decay with a small offset (red) introduced by noise over the small field range included in the fit. Rate constants provided in Figure 4C in main text.

trEPR of GdDM

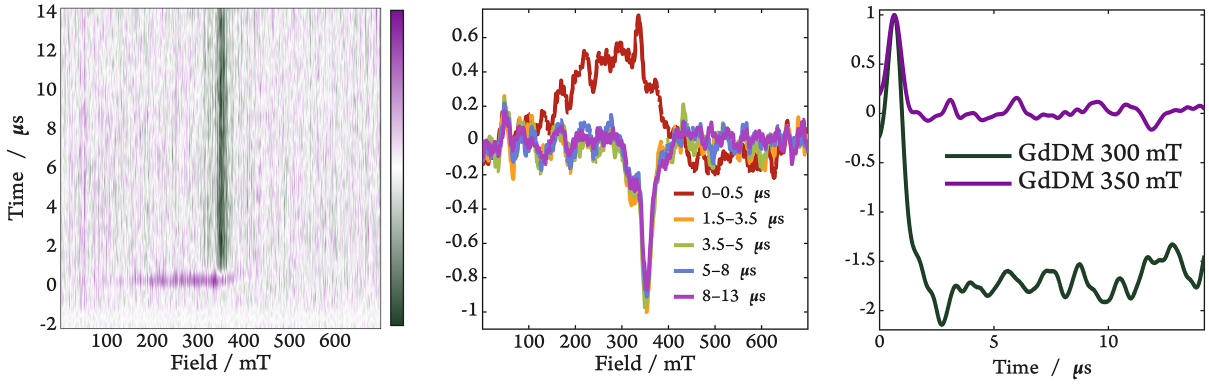

Figure S12: **GdDM**, trEPR, 200 K, EKSPLA. Left: Time- and field-resolved data. Right: Selected time slices of trEPR spectra. Decay of the broach signal at 300 mT (purple) and sharp signal at 350 mT (green).

The trEPR signal intensities of both **H_3DM**, Figure S10, and **LuDM**, Figure S11, decay without change in spectral shape with respective lifetimes of 3.55 and 3.76 μs. However, after the rapid decay of the broad AE trEPR signal of **GdDM**, Figure S12 (purple signal in left and right panels), the sharp emissive species (green in left and right panels), centred around $g = 1.992$, exhibits only a minor decay in signal intensity during this time window.

80 K trEPR Data

We additionally recorded the trEPR data at 80 K to explore the impact of temperature on the lifetimes of all spin states contributing to the observed EPR spectra.

trEPR H_3DM

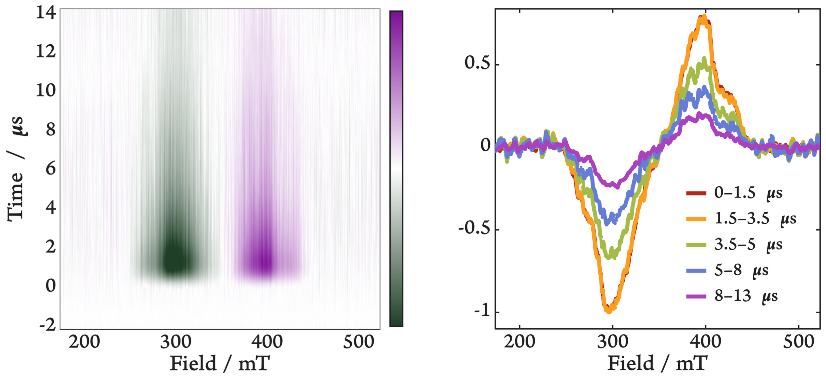

Figure S13: **H_3DM**, trEPR, 80 K, Opolette. Left: Time- and field-resolved data. Right: Selected time slices of trEPR spectra.

LuDM

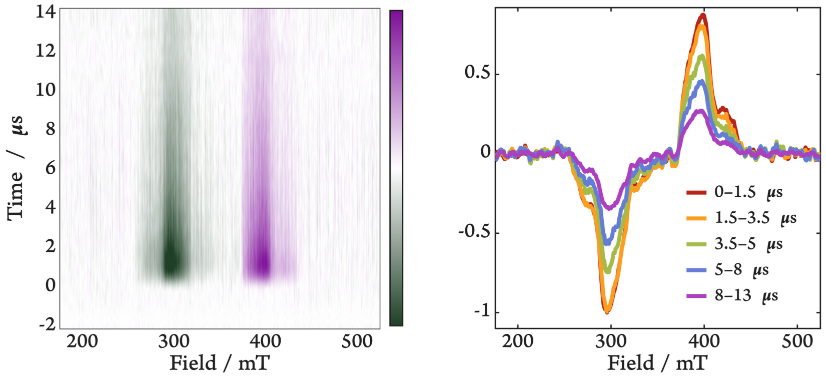

Figure S14: **LuDM**, trEPR, 80 K, Opolette. Left: Time- and field-resolved data. Right: Selected time slices of trEPR spectra.

GdDM

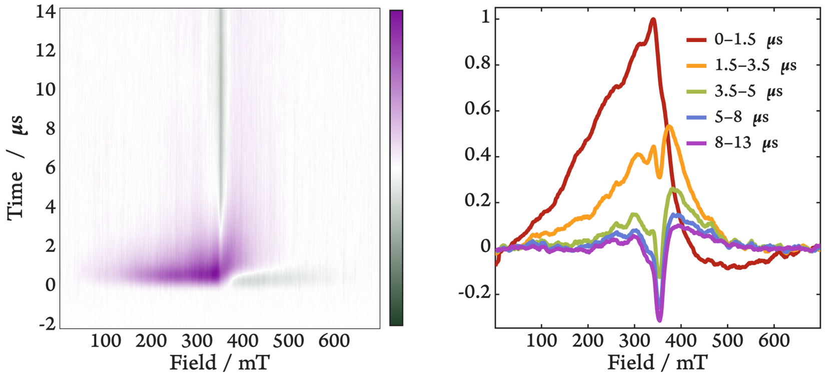

Figure S15: **GdDM**, trEPR, 80 K, EKSPLA. Left: Time- and field-resolved data. Right: Selected time slices of trEPR spectra.

Whilst somewhat slower as compared to 200 K, the trEPR signal intensities of both **H_3DM**, and **LuDM** decay significantly over 16 μs. In analogy with the diamagnetic analogues the broad signal in **GdDM** (purple signature in 2D panel) also decays somewhat slower at the lower temperature. However, the sharp emissive species (green in 2D panel), centred around $g = 1.992$, exhibits no visible decay of signal intensity in this time window. This, along with the data at 200 K, indicates this sharp emissive signal is not originating from the triplet state as seen in **H_3DM** or **LuDM** but is consistent with the involvement of the Gd^{3+} ground spin state whose spectral shape it resembles.

Additional trEPR Experiments

The trEPR signal of **GdDM** was also recorded: i) without the 3–300 kHz bandpass preamplifier (Figure S16), ii) following 410 nm laser excitation, iv) using a wider sweep width. No signal was observed in the region from 600–1200 mT at any time. No difference in spectral shape between excitation at 410 nm vs 355 nm. There was, also, no observed difference in spectral shape at lower LASER powers, just a reduced S/N in agreement in keeping with the UV/Vis data.

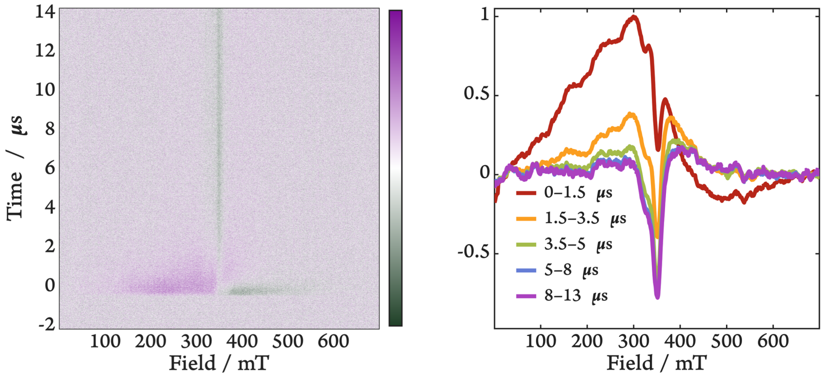

Figure S16: **GdDM**, 80K, 2 mJ, trEPR, EKSPLA, λ_{ex} = 410 nm. Left: Time- and field-resolved data. Right: Selected time slices of trEPR spectra. Data was recorded without a 3–300 kHz bandpass preamplifier. All other parameters are the same as in Figure S15.

Magnetophotoselection Experiments

Magnetophotoselection experiments were performed by placing a polariser, aligned either parallel or perpendicular to the external magnetic field, after the final turning mirror instead of a depolariser. The LASER power was carefully controlled so all measurement were performed with 2 mJ per shot. For **LuDM**, a GWU VersaScan/UVScan OPO LASER with a shot repetition rate of 20 Hz was used with λ_{ex} = 410 nm. For **GdDM**, a Continuum Surelight I Nd:YAG laser with a shot repetition rate of 10 Hz was used with λ_{ex} = 355 nm.

6. Density Functional Theory (DFT)

Previous work has suggested that there is at least one solvent molecule coordinated to the lanthanide ion.[1] All DFT calculations were performed using ORCA 5.1.2 and the PBE0 functional with the D3BJ dispersion correction.[2-6] Geometries were optimised as the glycerol adducts using DFT with the DEF2-TZVP basis set for light atoms and the ZARC-ZORA-TZVP SARC/J basis set for lanthanide atoms.[7-11] EPR calculations were performed using the EPR-II basis set for light atoms and the ZARC-ZORA-TZVP basis set for lanthanide ions.[12] The geometry of both the **LnDM** complexes were optimised as the water and glycerol adduct. Frequency calculations were performed to confirm there were no imaginary frequencies. Whilst DFT only reliably provides qualitative values for binding energies, EPR calculations were performed as the glycerol adduct as DFT indicated this was significantly more thermodynamically favourable, Table S1, than the H_2O adduct. No spin-orbit coupling was included in the EPR calculations as there was negligible delocalisation of spin density onto any heavy atoms, Figure S18.

Table S1: Predicted Gibbs Free Energy (G) of relevant species.

**Species**	**Predicted G (Eh)**
**LuDM**	-16796
**LuDM**.H_2O	-168723
**LuDM**.Glycerol	-17141
H_2O	-76
Glycerol	-344

Equation S1: ΔG binding energies for water and glycerol to **LuDM**.

$$\Delta_{\text{bind}}G(\text{H}_2\text{O}) = -255 \text{ kJ } mol^{-1}$$
$$\Delta_{\text{bind}}G(\text{Glycerol}) = -858 \text{ kJ } mol^{-1}$$

a. Spin Density Plots

Spin densities were generated as a Gaussian Cube file directly from the EPR calculation. The spin densities were visualised using UCSF ChimeraX, developed by the Resource for Biocomputing, Visualization, and Informatics at the University of California, San Francisco, with support from National Institutes of Health R01-GM129325 and the Office of Cyber Infrastructure and Computational Biology, National Institute of Allergy and Infectious Diseases.) [13]

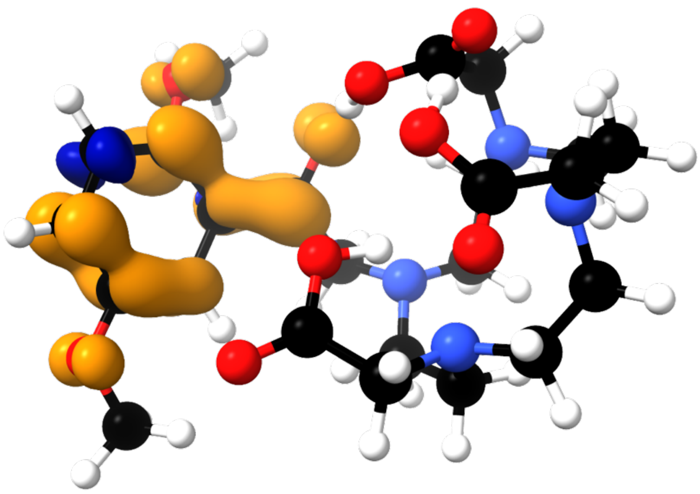

Figure S17: Spin density of **H_3DM** T_1 excited state

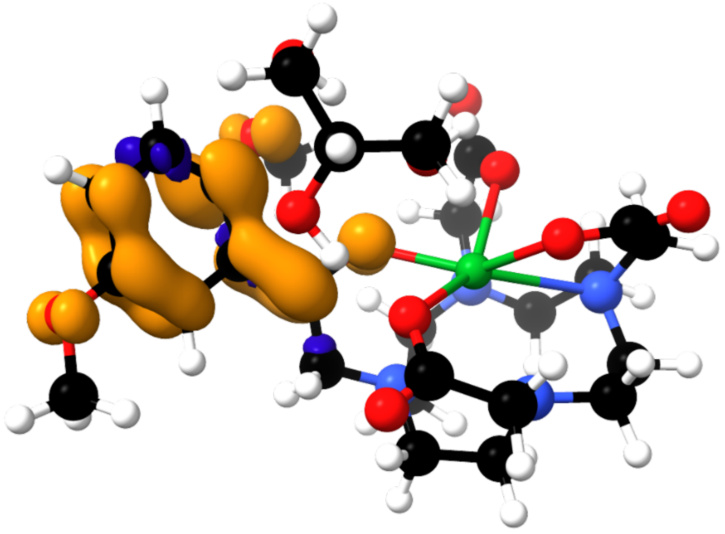

Figure S18: Spin density of **LuDM** of T_1 excited state

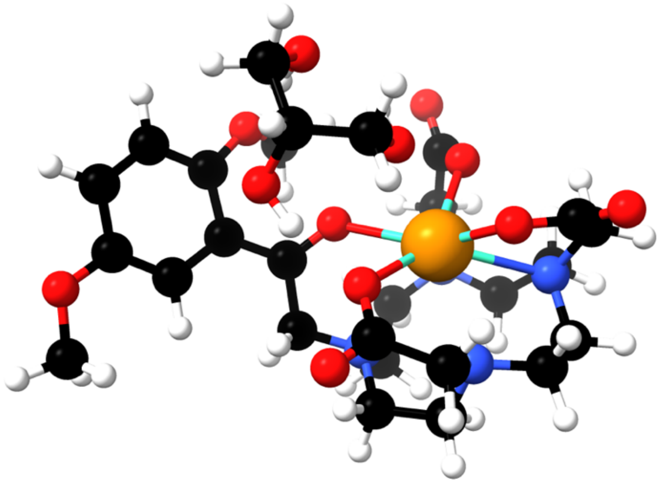

Figure S19: Spin density of **GdDM** $^8S_{7/2}$ ground state

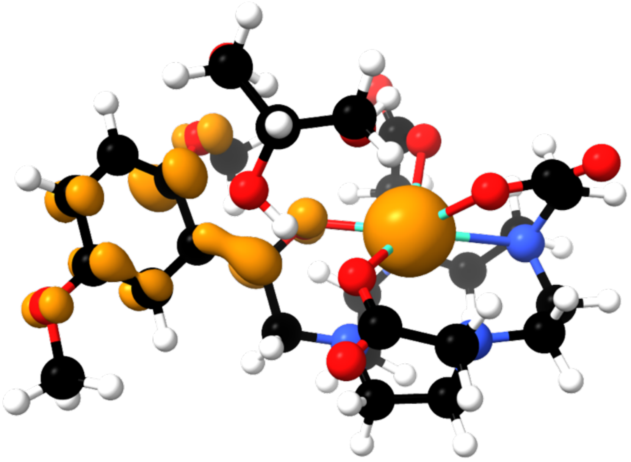

Figure S20: Spin density of **GdDM** $\{T_1-{}^8S_{7/2}\}$ excited state.

7. Spectral Simulations

trEPR spectra were simulated using MATLAB (R2022b) as absorption powder spectra using full matrix diagonalisation via the pepper function from the EasySpin (v. 6.0) package.[14-17] DFT was used to estimate initial parameters, from which bounded simulation fitting was performed to achieve reasonable simulations.

a. H_3DM

Table S2: Comparison between predicted values (DFT) and values extracted from fitting spectrum averaged between 0.8–1.8 μs after laser pulse for **H_3DM**

Parameter	DFT	Simulation
g (xyz)	2.002 2.004 2.005	1.996 1.996 2.001
D / MHz	- 1868	- 2467
E / MHz	400	293

b. LuDM

Table S3: Comparison between predicted values (DFT) and values extracted from fitting spectrum averaged between 0.8–1.8 μs after laser pulse for **LuDM**

Parameter	DFT	Simulation
g (xyz)	2.002 2.005 2.005	1.994 1.993 1.994
D / MHz	- 2704	- 2336
E / MHz	278	304

b. GdDM

Simulation Procedure

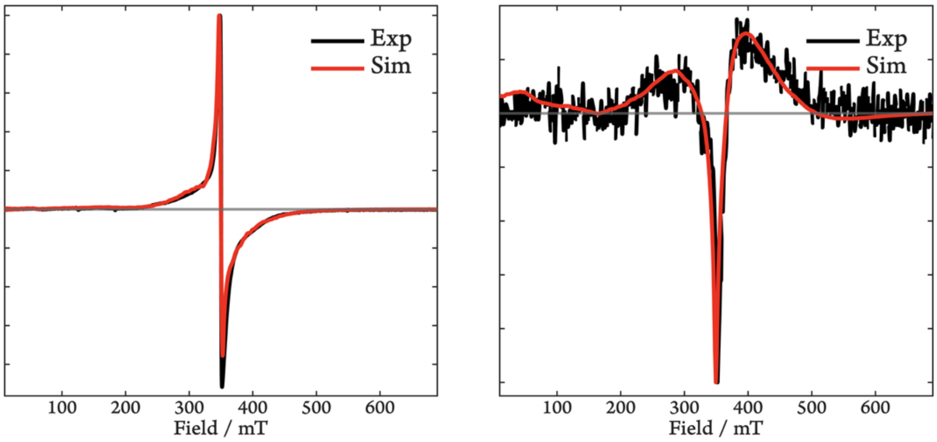

Figure S21. Left: Experimental cwEPR spectrum of **GdDM** (black) and a fitted spectrum (red) at 80 K utilising the method outlined by Clayton et al. [18] The g-value was set at 1.992 and values for the centre of the distributions at ± D_0 = 1008 MHz and the full width half max, D_{fwhm} = 851 MHz, of the distribution could be obtained from the cwEPR spectrum. Right: Experimental trEPR spectrum of **GdDM** (black), averaged between 7–11 μs after the laser flash at 80 K, and a fitted spectrum (red). The fitted spectrum was simulated by using the same values for D_0 and D_{fwhm} as the ground state cwEPR spectrum and only adjusting both the polarisations of the Gd^{3+} sub-levels and the ratio, ASP, between the distributions centred at ($+D_0$:$-D_0$). The following polarisation of the Gd^{3+} sub-levels in the eigenbasis, in increasing energetic order: [0.309 0.199 0.005 0.000 0.050 0.096 0.004 0.338] and the ASP = 0.125 could then be obtained.

For **GdDM**, the EPR spectra were fitted utilising a method outlined by Clayton et al., which is an adaptation of the method initially proposed by Raitsimring et al.[18, 19]. This required the explicit sampling of a bimodal Gaussian distribution of the zero-field splitting parameter, D, to account for the large anisotropic broadening, caused by the large D-Strain, typically seen in EPR spectra of Gd^{3+} ions. The method utilises an isotropic **g**-tensor with g_{iso} = 1.992 and could be used to estimate both the centre of the Gaussian distributions at ± D_0 and the full width half max, D_{fwhm}, of the distribution. The distribution was sampled with 1200 points between - 3D_0 and + 3D_0. Clayton et al. showed that the inclusion of an asymmetry parameter, ASP, which is the ratio of the intensities of the Gaussian distributions centred at ($+D_0$:$-D_0$), was necessary to account for the asymmetry in spectra only at high fields (G-band) where there are large polarisation differences. The corresponding distribution of the other zero-field parameter, E, was accounted for by explicitly sampling 10 random points between $0 < E < D/3$ for each value of D. The weight, P(E/D) for each value E of given a value of D is given in equation S2.

Equation S2: Probability distribution of E/D

$$P\left({}^{E}/_{D}\right) = {}^{E}/_{D} - 2\left({}^{E}/_{D}\right)^{2}$$

The cwEPR spectrum was intially fitted, Figure S21 (left), to extract the values of D_0 and D_{fwhm}, Table S4, and are compared to other similar Gd^{3+} complexes in Table S6. The trEPR spectrum at late times (7–11 μs after laser flash) was then fitted, Figure S21 (right), using the same distribution for the **D**-tensor, extracted from fitting the cwEPR spectrum, and by adjusting both the polarisation of the Gd^{3+} sub-levels in the eigenbasis, Table S5, and the ASP, Table S11. The corresponding distribution for the **D**-tensor is shown in Figure S22.

Spin Hamiltonian Parameters

Table S4. Spin Hamiltonian parameters extracted from the fitting of the cwEPR spectrum of **GdDM** (X-Band, 80 K) using the method outlined by Clayton et al. [18]

Parameter	Value from cwEPR/trEPR simulation
D_0 (MHz)	1008
D_{fwhm} (MHz)	851
ASP	0.125

Table S5. Polarisation of Gd^{3+} sub-levels in the eigenbasis, in increasing energetic order, extracted from fitting the late-time (7–11 μs after laser flash) trEPR spectrum of **GdDM** (X-Band, 80 K) using the same values for D_0 and D_{fwhm} extracted from fitting the cwEPR spectrum in the dark.[18] In the high-field limit, these eigenstates are the [-7/2, -5/2, … , +7/2] m_s sub-levels.

Eigenstate	Polarisation from trEPR simulation
**1**	0.310
**2**	0.200
**3**	0.005
**4**	0.000
**5**	0.052
**6**	0.091
**7**	0.004
**8**	0.339

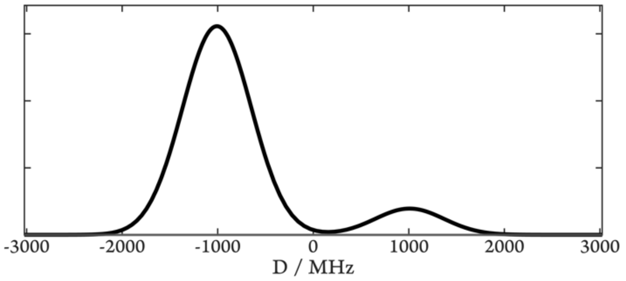

Figure S22. Distribution of the **D**-tensor: a bimodal Gaussian distribution centred at ± D_0 = 1008 MHz, with full width half max, D_{fwhm} = 851 MHz, and the ratio, *ASP*, of the amplitudes of the distributions centred at $+D_0$:$-D_0$, *ASP* = 0.125.

Evaluation of the Asymmetry Parameter

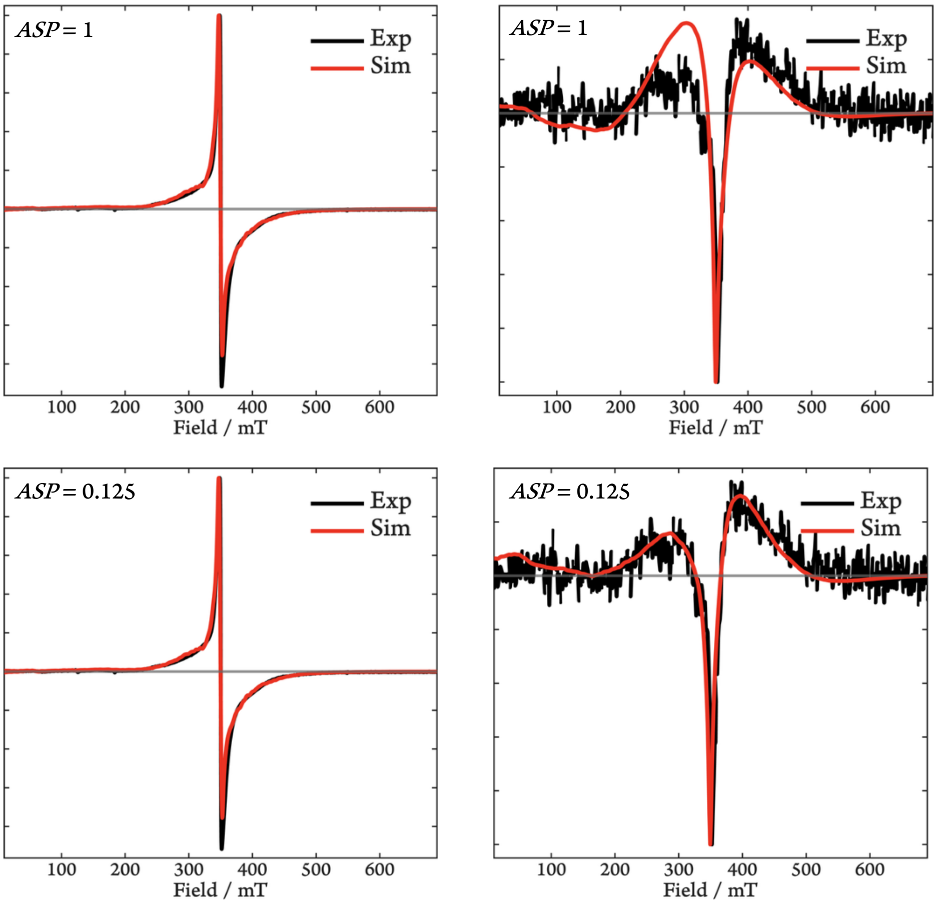

Figure S23: Comparison of the effect of including an *ASP* = 1 against an optimised value for the *ASP* = 0.125. All simulations follow the procedure described above and utilise the same spin Hamiltonian parameters, given in Table S4. The left-hand side shows the experimental cwEPR spectrum (black) in the dark with a simulated spectrum (red) included. The right-hand side shows the experimental trEPR spectrum (black) at late times, averaged between 7–11 μs after the laser flash, with a simulated spectrum (red) included, generated by only varying the polarisation of the Gd^{3+} sub-levels in the eigenbasis, as seen in Table S5. The top two panes have an *ASP* = 1 and the bottom two panes have the optimised value for the *ASP* = 0.125.

As can be seen in the left hand side of Figure S23, it was found the ratio of the amplitudes of the distributions centred at $(+D_0 : -D_0)$, denoted *ASP*, had a negligible effect on the fit of the cwEPR spectrum at X-Band, in line with the results of Clayton et al.[18] However, as can be seen by the right-hand side of Figure S23, the inclusion of an $ASP \neq 1$ was necessary to achieve a reasonable simulation of the experimental data probably due to the large optically induced spin polarisation of the Gd^{3+} ion, which makes the effect of the *ASP* more pronounced at lower microwave frequencies such as at X-Band used here.

Comparison to Other Similar Gd^{3+} ions

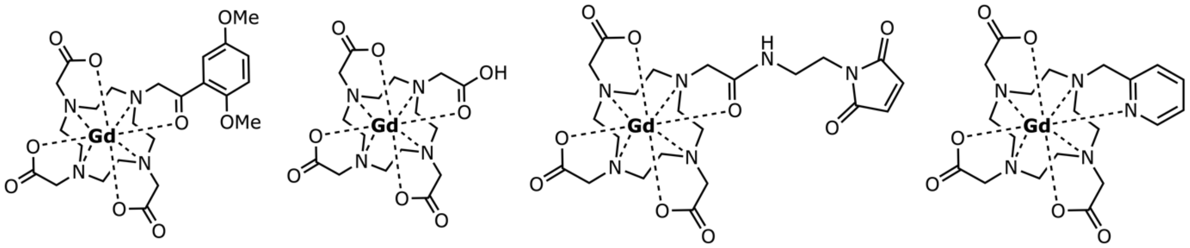

GdDM **Gd-DOTA** **Gd-maleimide-DOTA** **Gd-PyDTTA**

Figure S24: The range of similar gadolinium(III) complexes compared for their **D**-tensor distributions in Table S6.

It is clear from the values in Table S6 that the magnitude of the D_0 for **GdDM** is slightly larger than the other complexes, Figure S24, with an oxygen as their 8th donor atom, **Gd-DOTA** and **Gd-maleimide-DOTA**, but much smaller than the complex with a nitrogen as the 8th donor atom, **Gd-PyDTTA**. However, the system appears significantly

more strained than any of the other similar gadolinium(III) complexes investigated by Clayton et al. This is possibly due to the less well-defined coordination environment of the 2,5-dimethoxyacetphenone chromophore compared to the other 8th donor systems: carboxylic acid, secondary amide and pyridine for **Gd-DOTA**, **Gd-maleimide-DOTA** and **Gd-PyDTTA** respectively. The value for the *ASP* is less than one for all complexes, indicating a net-negative **D**-tensor. The smaller value for the *ASP* of **GdDM** is again potentially due to the less well-defined coordination geometry.

Table S6: Values for spin Hamiltonian parameters extracted from the fitting of the EPR spectra of **GdDM** compared to values for other similar gadolinium complexes, from Clayton et al.[18]

Parameter	GdDM cwEPR (dark)	Gd-DOTA	Gd-maleimide-DOTA	Gd-PyDTTA
D_0 (MHz)	1008	714	714	1830
D_{fwhm} (MHz)	851	328	328	390
ASP	0.13	0.30	0.30	3.60

Comparison of Light vs Dark Spectra

In order to compare the cwEPR in the dark and the trEPR spectrum with irradiation, a theoretical cwEPR spectrum, using the polarisations of the Gd^{3+} sub-levels in the eigenbasis, extracted from the trEPR simulation at late times, was generated. The linewidth and the modulation amplitude were set to the values for the **GdDM** cwEPR spectrum in the dark to generate the spectrum seen in Figure 5C in the main manuscript. The full width of the same plot is shown in Figure S25. As can be seen in Figure S25, the central transition, typically assigned to the $m_s = -1/2 \leftrightarrow +1/2$ transition in the high-field approximation (ZFS<<Zeeman) which is not entirely valid here (see Point 3 below), is inverted upon photoexcitation of the chromophore and it is obvious there is an effective hyperpolarisation of the Gd^{3+} sub-levels (as compared to Boltzmann).[19]

Note: Quantitative comparisons between Boltzmann distributed and spin polarised cases are not possible due to the following reasons:

1. Absolute populations of spin sub-levels can rarely be extracted from trEPR spectra. A notable exception to this rule is a system where the signal-to-noise ratio, optical and spin-lattice relaxation lifetimes allow the detection of the entire evolution of the spin polarised system from birth all the way to the thermal state. For most systems, including ours, this is not possible.
2. In a field-swept EPR spectrum of a dark state radical, the determination of absolute population differences is still problematic. At each point in the spectrum, the field is different, hence the Zeeman energy is different. Additionally, at each point in the spectrum, there is an overlap of resonances from molecules with different tensor orientations (primarily the **D**-tensor in this case). That is, at each field position there is a mixture of different molecules, different Hamiltonians, and different spin polarisations.
3. A final complication is that for our Gd^{3+} system, we have Zeeman/$D_0 \approx 9$. This means that there is only one orientation ($B||D_z$) where the eigenstates of the Hamiltonian are the same as the eigenstates of S_z and hence have a well-defined m_s = [-7/2 , …., +7/2]. This is not true for any of the other orientations. Therefore, labelling the states according to m_s is not correct.

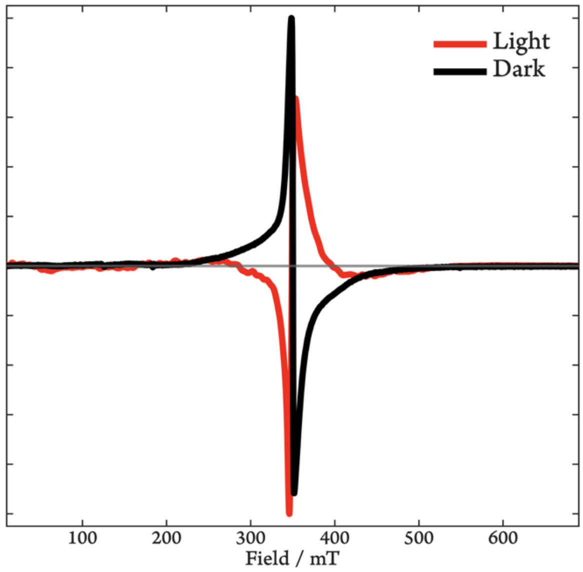

Figure S25. Experimental ground state cwEPR spectrum of **GdDM** (black) and the simulation (red) of a cwEPR spectrum employing the simulation magnetic parameters obtained for the photoinduced trEPR spectrum.

References

(1) Liao, Z.; Tropiano, M.; Mantulnikovs, K.; Faulkner, S.; Vosch, T.; Sorensen, T. J. Spectrally resolved confocal microscopy using lanthanide centred near-IR emission. *Chem Commun* **2015**, *51* (12), 2372–2375. DOI: 10.1039/c4cc09618e
(2) Neese, F. The orca program system. *Wiley Interdiscip Rev Comput Mol Sci* **2010**, *2*, 73–78. DOI: 10.1002/wcms.81.
(3) Neese, F. Software update: the orca program system, version 4.0. *Wiley Interdiscip Rev Comput Mol Sci* **2018**, *8*. DOI: 10.1002/wcms.1327.
(4) Neese, F.; Wennmohs, F.; Becker, U.; Riplinger, C. The orca quantum chemistry program package. *J Chem Phys* **2020**, *152*, 224108. DOI: 10.1063/5.0004608.
(5) Adamo, C.; Barone, V. Toward reliable density functional methods without adjustable parameters: The PBE0 model. *The Journal of Chemical Physics* **1999**, *110* (13), 6158–6170. DOI: 10.1063/1.478522
(6) Grimme, S.; Ehrlich, S.; Goerigk, L. Effect of the damping function in dispersion corrected density functional theory. *J Comp Chem* **2011**, *32* (7), 1456–1465. DOI: 10.1002/jcc.21759
(7) Schäfer, A.; Huber, C.; Ahlrichs, R. Fully optimized contracted Gaussian basis sets of triple zeta valence quality for atoms Li to Kr *J Chem Phys* **1994**, *100*, 5829–5835 DOI: 10.1063/1.467146
(8) Weigend, F.; Ahlrichs, R. Balanced basis sets of split valence, triple zeta valence and quadruple zeta valence quality for H to Rn: Design and assessment of accuracy. *Phys Chem Chem Phys* **2005**, *7* (18), 3297–3305. DOI: 10.1039/b508541a
(9) Weigend, F. Accurate Coulomb-fitting basis sets for H to Rn. *Phys Chem Chem Phys* **2006**, *8* (9), 1057–1065. DOI: 10.1039/b515623h
(10) Rolfes, J. D.; Neese, F.; Pantazis, D. A. All-electron scalar relativistic basis sets for the elements Rb-Xe. *J Comput Chem* **2020**, *41* (20), 1842–1849. DOI: 10.1002/jcc.26355
(11) van Lenthe, E.; Snijders, J. G.; Baerends, E. J. The zero-order regular approximation for relativistic effects: The effect of spin–orbit coupling in closed shell molecules. *The Journal of Chemical Physics* **1996**, *105* (15), 6505–6516. DOI: 10.1063/1.472460
(12) Barone, V. *Recent Advances in Density Functional Methods*; World Scientific Publ. Co, 1996.
(13) Meng, E. C.; Goddard, T. D.; Pettersen, E. F.; Couch, G. S.; Pearson, Z. J.; Morris, J. H.; Ferrin, T. E. UCSF ChimeraX: Tools for structure building and analysis. *Protein Sci* **2023**, *32* (11), e4792. DOI: 10.1002/pro.4792
(14) Stoll, S.; Schweiger, A. EasySpin, a comprehensive software package for spectral simulation and analysis in EPR. *J Magn Reson* **2006**, *178* (1), 42–55. DOI: 10.1016/j.jmr.2005.08.013
(15) Stoll, S. Computational Modeling and Least-Squares Fitting of EPR Spectra. In *Handbook of Multifrequency Electron Paramagnetic Resonance: Data and Techniques*, Misra, S. K. Ed.; Wiley-VCH, 2014.
(16) Stoll, S. CW-EPR Spectral Simulations: Solid State. *Methods Enzymol* **2015**, *563*, 121–142. DOI: 10.1016/bs.mie.2015.06.003
(17) Tait, C. E.; Krzyaniak, M. D.; Stoll, S. Computational tools for the simulation and analysis of spin-polarized EPR spectra. *J Magn Reson* **2023**, *349*, 107410–107425. DOI: 10.1016/j.jmr.2023.107410
(18) Clayton, J. A.; Keller, K.; Qi, M.; Wegner, J.; Koch, V.; Hintz, H.; Godt, A.; Han, S.; Jeschke, G.; Sherwin, M. S.; Yulikov, M. Quantitative analysis of zero-field splitting parameter distributions in Gd(iii) complexes. *Phys Chem Chem Phys* **2018**, *20* (15), 10470–10492. DOI: 10.1039/c7cp08507a
(19) Raitsimring, A. M.; Astashkin, A. V.; Poluektov, O. G.; Caravan, P. High-Field Pulsed EPR and ENDOR of Gd(3+) Complexes in Glassy Solutions. *Appl Magn Reson* **2005**, *28*, 281–295. DOI: 10.1007/BF03166762
